# Biosensors for Stress Detection: A Systematic Review from Herbaceous to Woody Plants

**DOI:** 10.3390/bios16050242

**Published:** 2026-04-25

**Authors:** Raffaella Margherita Zampieri, Alessandro Bizzarri, Eleftherios Touloupakis, Serena Laschi, Ilaria Palchetti, Claudia Cocozza, Alessio Giovannelli

**Affiliations:** 1 Department of Agriculture, Food, Environment and Forestry (DAGRI), University of Florence, Piazzale delle Cascine 18, 50144 Florence, Italy; raffaellamargherita.zampieri@unifi.it (R.M.Z.); alessandro.bizzarri@unifi.it (A.B.); claudia.cocozza@unifi.it (C.C.); 2 Research Institute on Terrestrial Ecosystems (IRET), National Research Council (CNR), Via Madonna del Piano 10, 50019 Sesto Fiorentino, Italy; alessio.giovannelli@cnr.it; 3 Department of Chemistry “Ugo Schiff” (DICUS), University of Florence, Via della Lastruccia 3, 50019 Sesto Fiorentino, Italy; serena.laschi@unifi.it (S.L.); ilaria.palchetti@unifi.it (I.P.)

**Keywords:** abiotic stress, biotic stress, sensor, forestry, agriculture, plant health, plant vitality, fluorescent probe, electrochemical sensor

## Abstract

Plants must constantly adapt to biotic and abiotic stressors, which the global climate change crisis has intensified. To monitor plant health and predict their ability to face these challenges, various target molecules, such as hormones, glucose, and reactive oxygen species, are used as proxies for their physiological status. This review provides a systematic assessment of the current state of biosensor technology, an innovative analytical approach designed for in situ, minimally invasive, and real-time monitoring. Using the PICO (Problem, Intervention, Comparison, and Outcome) strategy, relevant research papers were identified. The review highlights how biosensors can detect physiological responses to stress before visual symptoms manifest, offering a significant advantage over traditional, often destructive, laboratory techniques, like gas chromatography–mass spectrometer (GC-MS) or high-performance liquid chromatography (HPLC). These advancements aim to improve precision agriculture and forestry management by providing sustainable methods to assess resilience in changing environments. Finally, the challenges of translating research from model organisms to complex woody species and choosing the correct target are discussed, and future perspectives, including the integration of biosensors with Artificial Intelligence-driven predictive models for large-scale environmental monitoring, are outlined.

## 1. Introduction

The global climate crisis has intensified the frequency and severity of stress, pushing many plant species to their physiological limits. Plants, being sessile organisms, have evolved adaptive mechanisms to cope with environmental challenges. However, events such as extreme temperature fluctuations, prolonged droughts, and irregular precipitation patterns pose a significant threat to plant health, survival and productivity. Understanding how plants respond to stress and monitoring their development is fundamental to reducing irreversible damage and yield loss. Thus, there is an urgent need for advanced analytical tools capable of monitoring plant status in real-time, allowing researchers to unravel the physiological signatures of stress before visual symptoms manifest. The concept of stress itself, applied in plants, can arguably be difficult to establish: their modular structure, where tissues operate with varying degrees of independence, makes it challenging to define a unified stress response. This complicates the understanding of how the whole plant reacts to a highly variable environment [1]. As climate change accelerates, stressors can reach levels that were unprecedented during the period used to calibrate predictive models. Indeed, the relationship between environmental stressors and tree growth is fundamentally non-linear, meaning that a response to a climatic variable does not produce a proportional, constant change in plant growth across all conditions. This non-linearity is a primary driver of non-stationarity, where the historical relationship used to model tree growth fails to predict future growth as climate conditions shift [2].

Traditional methodologies for assessing plant stress are generally ineffective for high-throughput, real-time monitoring. Gold standard techniques, such as GC-MS, HPLC, molecular methods (PCR, Polymerase Chain Reaction), biochemical assays, and immunoassays (ELISA, Enzyme-Linked ImmunoSorbent Assay), while accurate, require complex sample preparation, expensive instruments and reagents, and trained personnel [3]. Moreover, the equipment needed can hardly be applied to field studies. These approaches are inherently time-consuming, laborious, and can preclude the possibility of monitoring the same individual plant over a continuous timeline if the protocol imposes destructive sampling. Lastly, in the context of studying stress response and tolerance, it is crucial to remember that these are highly dependent on the environment [4]. Studies performed in controlled conditions, often focusing on single stress effects and indoor conditions, do not reflect the complexity of the field, where multiple biotic and abiotic stressors are combined. Adding to the complexity, plant physiological responses to environmental stressors are fundamentally non-linear, characterized by complex feedback loops, threshold effects, and hormetic responses [5]. Unlike proportional linear models, plant fitness often follows a biphasic dose–response curve where low-level stressors stimulate compensatory pathways, while exceeding critical intensity or duration thresholds can determine a decline in metabolic function. Predicting plant plasticity in fluctuating environments requires an integrated understanding of these thresholds, where minor incremental changes in a stressor can lead to disproportionate shifts in plant health and productivity. Consequently, there is a need for non-destructive and integrated tools that can bridge the gap between analytical precision and ecosystem-scale monitoring.

According to the International Union of Pure and Applied Chemistry (IUPAC) technical reports [6,7], a biosensor is an analytical device that integrates a biological (or biomimetic) recognition element (e.g., enzymes, antibodies, nucleic acids) with a physicochemical transducer (electrochemical, mass, magnetic, optical, or thermal transducer). Thus, biosensors belong to the broader family of chemical sensors. The transducer converts the biorecognition event into analytically useful information, such as analyte concentration, chemical structure, or changes in chemical structure [8]. Biosensors present many advantages: measurements are fast, usually taking no more than a few minutes, and can be multiplexed, i.e., allowing the simultaneous determination of different analytes [9,10]. When continuous monitoring is required, real-time applications can be developed, with limiting factors that should be considered, such as the affinity of the biological element with the target molecule and the degradation of the biological element itself [11]. A biosensor should present high selectivity for the analyte of interest, which has to be detected in a mixture of molecules and in the presence of unwanted contaminants with minimal sample treatment. Furthermore, high sensitivity is desirable, as a steep response (i.e., a high slope of the calibration curve) ensures that even small changes in analyte concentration result in significant and measurable changes in the output signal [12]. Moreover, measurements are usually highly reproducible and accurate [13]. Finally, biosensors can be implemented in systems that can be used in the field by farmers and forest management personnel. Many biosensors can represent a reliable low-cost analytical approach for screening analysis, an alternative to expensive laboratory techniques, where, on the other hand, an investment in equipment, trained and professional personnel and chemicals is generally required.

All these advantages make the application of biosensors a promising tool to efficiently assess the physiological status of plants. Given the accuracy and timeliness of the measurements, biosensors could ameliorate the response obtained using conventional procedures regarding the physiology of the plant and the damage caused by stressors. For instance, visual assessments, though non-invasive, fast, and cost-effective, are inherently subjective and often identify stress only until visible morphological changes, such as chlorosis or wilting, or after irreversible physiological damage have occurred [14]. For this reason, their utilization could help sector experts or employees create a device for prompt and precise decision-making, with important benefits in risk management.

As climate change continues to reshape environmental landscapes, plants are recurrently exposed to suboptimal conditions that compromise their growth and survival [15]. Detecting and quantifying these stress responses is critical for both ecological conservation, restoration actions and crop management. Given the complexity of plant–environmental interactions, the development of precise tools to evaluate plant health status has become a keystone of contemporary research, providing the data necessary to navigate the challenges of a warming world.

In the past, stressors were often studied in isolation. However, global change acts as a threat multiplier, creating a lethal synergy between different types of pressure: abiotic (heatwaves, prolonged droughts, pollution) and biotic stress (invasive insects, fungi, and bacteria that migrate to new areas as temperatures rise) [16]. Developing biosensors essentially turns plants into environmental sentinels, providing real-time feedback for the health of the ecosystem. By understanding physiological responses, we can select specific genotypes or species capable of maintaining homeostasis under extreme conditions, ensuring that newly planted forests survive into the next century. Long-term monitoring of these plants, which could be performed using this innovative technology, will fill the gap in the effectiveness of ecological restoration programs.

The aim of this review is to identify the state of the art of biosensor development and applicability to plant stress evaluation, considering both herbaceous and woody species to highlight the technological transition from short-lived model organisms to complex vascular perennials. Therefore, perspectives are given on how new biosensors should be developed and why they could function as a valuable tool to assist agroforestry services.

## 2. Research of Scientific Articles According to the PICO Strategy

The review was conducted in a systematic manner according to the PRISMA (Preferred Reporting Items for Systematic reviews and Meta-Analyses) 2020 statement [17]. The abstract checklist and the PRISMA checklist are available in the Supplementary Materials (Tables S1 and S2, respectively). The PICO (Problem, Intervention, Comparison, and Outcome) strategy was applied to perform the bibliographical search and select relevant papers [18]. Two research processes were conducted in parallel for herbaceous and woody plants, with the four parameters defined as follows: the problem was the early detection of stress in plants; the intervention was the use of biosensors, compared with analytical and laboratory analysis; and the outcome was the identification of any biosensor developed for the molecule’s detection. Scopus and Web of Science were used as databases. A thorough description of the selection process is reported in Appendix A.

**Figure 1:**
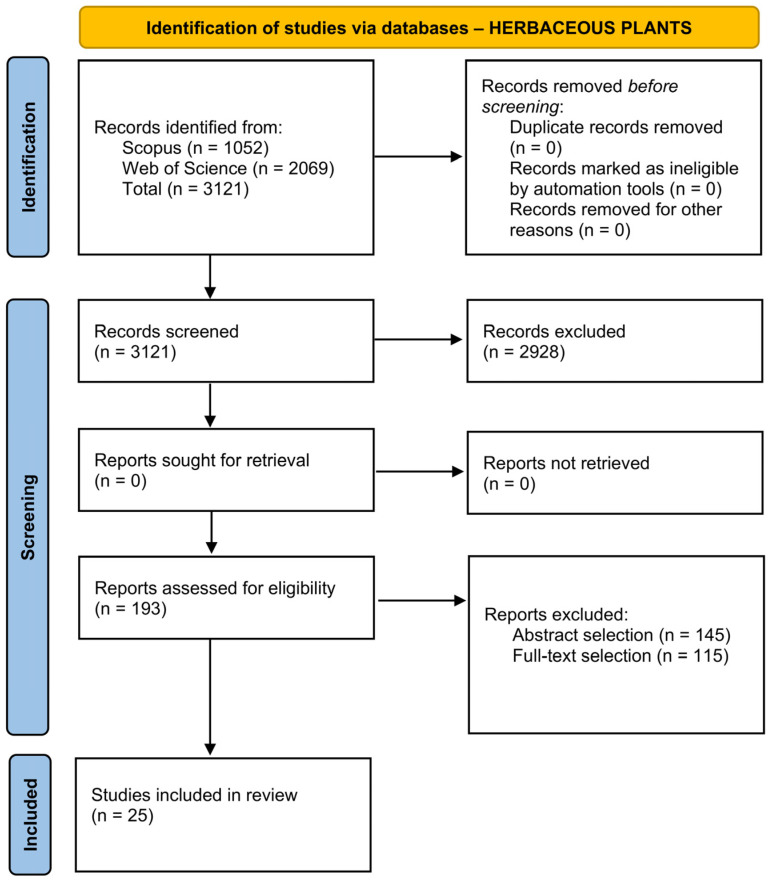
PRISMA diagram for the selection of research papers considered in this review for herbaceous plants.

**Figure 2:**
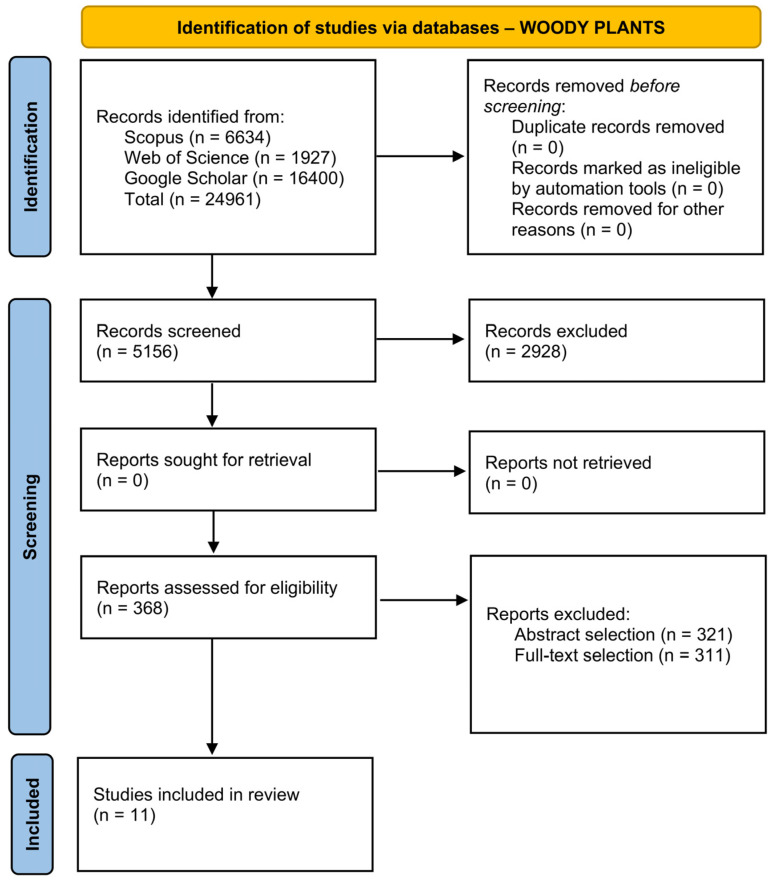
PRISMA diagram for the selection of research papers considered in this review for woody plants.

It is interesting to note that, despite filtering by research papers, most of the results obtained were reviews, making the screening process more time-consuming than expected. Research papers that did not fall into the strict requirements selected for the research process were excluded. The process resulted in 36 research papers, 25 for herbaceous plants (Figure 1) and 11 for woody plants (Figure 2).

Articles in which biosensors were developed and applied to plants, but did not present a stress-related scope, were not considered. The inferior number of articles in the woody plant section is a result of their lower availability, rather than a consequence of the research process. Additionally, many research papers found using the selected keywords do not fall within the definition of a biosensor. Indeed, a major problem noted during article screening is the lack of clarity in the use of the term biosensor. The most common definition, described in the Introduction and reported in the IUPAC technical reports and *Gold Book*, is “A device that uses specific biochemical reactions […] to detect chemical compounds usually by electrical, thermal or optical signals” [7]. Examples of misuse of the word are related to chemical sensors that do not present a biological (or biomimetic) component, even though the measure is applied to a biological matrix; physical sensors; the analysis of the microbial population as index of the physiological status of an organism, through common sequencing techniques; the use of organisms (e.g., plants) and their phenotype for assessing the level of pollution in the environment; the use of remote sensing technologies (e.g., LiDAR, Light Detection And Ranging; UAV, Unmanned Aerial Vehicle), despite the target being forests or fields. Articles focusing on Artificial Intelligence (AI)-driven data analysis were excluded if the development of the biosensor itself was not the primary focus of the study. Lastly, the PICO research was set up with the goal of screening the available literature for three pillars: plants, stresses, and biosensors. As a consequence of this decision, it is possible that the number of articles regarding the fluorescent probe section is limited by the term biosensor itself. However, a general highlight of the state of the art in this specific sector is given.

## 3. Biosensors to Detect Stress in Herbaceous Plants

As a result of the strict screening process, 25 articles on herbaceous plants were obtained and categorized according to the transducer type: electrochemical (Table 1) or fluorescence-based optical biosensors (Table 2 and Table 3).

### 3.1. Electrochemical Biosensors

By utilizing specific enzymes or probes, electrochemical biosensors can precisely detect substances, such as hormones, hydrogen sulfide (H_2_S), hydrogen peroxide (H_2_O_2_), glucose, and microRNA (Table 1). The studies used a variety of agricultural and model species, such as corn, soybean, rice, tomato and *Arabidopsis thaliana*. Additionally, the biosensors were tested on horticultural and ornamental plants, including lettuce and gerbera.

Among the target molecules considered, indole-3-acetic acid (IAA), the most physiologically active form of the hormone auxin, is considered a critical indicator of plant stress because it regulates essential developmental processes that are directly impacted by environmental conditions [19]. Under water and salt stress, plants may experience an overaccumulation of IAA that inhibits root and shoot growth. For instance, amperometric biosensors were developed using nanomaterial-enhanced platinum microelectrodes for the direct quantification of IAA in raw plant tissue extracts [19]. Abscisic acid (ABA) is a marker of plant status for its indispensable role in abiotic stress responses (stomatal regulation) and physiological signal transduction. As an endogenous hormone, it acts as a primary mediator enabling plants to face drought, low temperature, and salt stress. A biosensor based on localized surface plasmon resonance was developed using aptamer-functionalized gold nanoparticles to detect ABA through conformational changes and nanoparticle aggregation [20].

Aside from hormones, glucose, being the primary energy source of cells and a central byproduct of sucrose metabolism and cell wall formation, can reveal the physiological status of plants [21]. Beyond its role as a nutrient and base molecule of starch and cellulose structures, it acts as a signaling molecule and molecular modulator that regulates biological processes ranging from germination to senescence [22]. When a plant is subjected to a significant stressor, such as wildfire, the concentration and movement of glucose within its tissues undergo several critical changes that indicate the plant’s level of vitality or damage. The primary impact of physical stress, like fire, is the disruption of the phloem and cambium; translocation of photosynthates, rich in glucose, is impaired or stopped entirely. Enzymatic machinery can be inhibited, preventing the conversion of stored starch back into usable glucose for recovery. If stress prevents the plant from transporting or utilizing its glucose effectively, it can lead to carbon starvation, one of the primary drivers of tree mortality. Indeed, in cases where stress is lethal, glucose levels may drop until they are no longer detected by laboratory equipment [23]. Applications include an integrated glucose monitoring system that utilizes 3D-printed polymer hollow microneedles housing a platinum wire with glucose oxidase (GOX) as a sensing interface, for minimally invasive detection [24]. A wearable electrochemical glucose-selective platform was developed using reverse iontophoresis to extract metabolites non-invasively through the leaf surface, secured by a magnetic sandwich-clamp [22]. A low-cost device was developed combining 3D-printed hollow microneedles and a biosensor based on screen-printed technology to collect fluid for the electrochemical analysis of glucose, pH, and H_2_O_2_ [25].

A biosensor utilizing a biocompatible biohydrogel (chitosan and reduced graphene oxide) was integrated into a microneedle array for rapid in situ monitoring of this hydrogen peroxide (H_2_O_2_), a reactive oxygen species (ROS) [26]. H_2_O_2_ is highly responsive to environmental changes, with its production significantly impacted by pathogen infections, drought, temperature extremes, salinity, and mechanical injuries [26]. Real-time kinetics of H_2_O_2_ generation were monitored at the sub-cellular level, specifically within photosystem II membranes, using an amperometric biosensor featuring an osmium-horseradish peroxidase (HRP) mediator [27]. Then, a disposable biosensor for its quantification was constructed using laser-induced graphene modified with gold particles and the enzyme sulfide: quinone oxidoreductase to monitor endogenous levels of hydrogen sulfide (H_2_S) in protoplasts [28]. H_2_S is a regulator involved in seed germination, plant development, regulation of cellular metabolism, and response to environmental stimuli [28].

Biosensors described are specifically designed for and applied to in vivo measurements, allowing for the real-time monitoring of plant health directly on the living organism. These devices often utilize wearable or minimally invasive designs to capture physiological data without destroying plant tissue. The development of in vivo sensors shifts from point measurements with laboratory analysis to continuous, on-site monitoring that mimics wearable medical devices for humans.

**Table 1:** Characteristics of electrochemical biosensors applied to herbaceous plants.

Plant Species	Plant Tissue	Biosensor	Target	Stress	Reference
Corn (*Zea mays* L. subsp. mays B73)	Root and shoot extracts	Amperometric, platinum disks with silanization and multiwalled carbon nanotubes	IAA	Abiotic (salinity: 100, 150, and 200 mM NaCl; water stress: −3, −6, and −9 bars of water potential created using polyethylene glycol)	[19]
Spinach (*Spinacia oleracea* L.)	Photosystem II membranes	Amperometric, carbon electrode with osmium and HRP	H_2_O_2_	Abiotic (high light: 1000 μmol photons m^−2^s^−1^)	[27]
Rice (*Oryza sativa* L.)	Fresh leaves extract	Localized surface plasmon resonance- based with abscisic acid aptamer and gold nanoparticles	ABA	Abiotic (drought, low temperature, high salinity)	[20]
Soybean (*Glycine max* L.)	Protoplasts (in vivo)	Glassy carbon electrode with Anti-Immunoglobulin-Au, Anti-vitronectin-like protein, cysteine	Vitronectin-like protein	Abiotic (heavy metal: lanthanum and lead)	[29]
*Capsicum annuum* L., *Gerbera* *jamesonii*, *Lactuca sativa* L.	Leaves (in vivo)	Ionotophoretic electrode with screen-printed-based system, polyvinyl alcohol, Prussian Blue, GOX	Glucose	Abiotic (low and high temperature 10, 25 and 40 °C; low light; different light spectra)	[22]
Tomato (*Solanum lycopersicum* L.)	Stem and leaves (in vivo)	hollow microneedle system with Nafion, GOX, gold nanoparticles	Glucose	Abiotic (salinity: 200 mM NaCl)	[24]
*Pilea pepermioides*, *Curio rowleyanus*, *Zamioculcas zamiifolia*, *Echeveria* Raindrops, *Alocasia* Yucatan Princess	Leaves (in vivo)	3D-printed hollow microneedle arrays patches with screen-printed electrodes, GOX, Prussian Blue, polyaniline	H_2_O_2_, glucose, pH	Biotic and abiotic	[25]
*Arabidopsis thaliana* L.	Protoplasts	Laser-induced graphene electrode with sulfide: quinone oxidoreductase, dihexadecylphosphate, gold particles	H_2_S	Abiotic	[28]
Soybean (*Glycine max* L. cv. Williams 82), Tobacco (*Nicotiana tabacum* L. cv. Xanthi nc)	Leaves extract	Microneedle sensor and gold-based electrode with HRP, chitosan, reduced graphene oxide	H_2_O_2_	Biotic (inoculation of *Pseudomonas syringae* pathovar *tomato* DC3000)	[26]
Rice (*Oryza sativa* L.)	RNA from leaves of 21-day-old seedlings	Screen-printed carbon electrodes with palladium and gold nanoparticles, violet phosphorene, single-stranded DNA, HRP	microRNA-319	Abiotic (drought, high temperature, salinity)	[30]

### 3.2. Optical Biosensors Using Injected Fluorescent Probes

An injected fluorescent probe acts as a biosensor by utilizing specific chemical or physical interactions within the plant’s internal environment to produce a measurable optical signal (Table 2). These probes are widely used as nondestructive visualization tools for studying biological functions. They are typically added through syringe infiltration into the apoplast, where they respond to stressors like drought, salinity, or pathogens. Some probes are designed to bind specific stress-related proteins; for example, flubactin (*N*-(2,5-dimethoxyphenyl)-5-dansylamide) was developed to target the ABA receptor PYR1 (Pyrabactin Resistance 1). This interaction increases fluorescence intensity, enabling real-time and in vivo monitoring of salt stress in plants [31]. Targeting the PYR1 receptor, an innovative agricultural biosensor was developed utilizing genetically engineered bacteria, specifically *Escherichia coli*, to function as a molecular signal transducer within plants. The detection system utilizes a luciferase enzyme that triggers a luminescent signal upon the binding of ABA to its receptor [32]. Probes can also act as sensors by undergoing chemical transformations in response to the accumulation of signaling molecules like H_2_O_2_. When H_2_O_2_ levels rise due to abiotic stress (e.g., wounding, high salinity, drought), the probe is oxidized, leading to fluorescence quenching visible to the naked eye under UV light, providing a simple indicator of plant health status [33]. In some biosensor designs, the probe is part of a larger system directly integrated into plants. For example, a lateral flow technology made of antibody-functionalized microspheres, injected within leaf tissue, allows analyte capture and signal generation. The capture and detection of the target analyte by the antibody-functionalized microspheres are concentration-dependent [34]. The pH acts as a signaling molecule and secondary messenger, regulating responses to stress like drought, salinity, and pathogens by altering protein/enzyme activity and triggering downstream cascades. Biosensors in the form of fluorescent proteins can be injected with a syringe to map these changes. For instance, a fluorescent reporter protein derived from *Ptilosarcus gurneyi* (Pt-GFP, green fluorescent protein) is exploited for its responsiveness to proton (H+) concentration in its immediate environment [35]. The sensor can quantify pH according to the ratio of its emission intensities when stimulated by two different light wavelengths.

These biosensors offer many advantages, including real-time monitoring of live plants in the field; minimal invasiveness, as they utilize the plant’s stomata or simple syringe pressure for probe uptake; and finally, independence from genetic transformation, allowing researchers to use advanced sensors in crop plants that are difficult to transform genetically.

**Table 2:** Features of injected fluorescent probes as optical biosensors.

Plant Species	Plant Tissue	Biosensor	Target	Stress	Reference
*Vicia faba* L., *Avena sativa* L.	Leaves (in vivo)	Fluorescent pH reporter protein from *Ptilosarcus gurneyi* (*Pt*-GFP)	pH	Biotic and abiotic	[35]
Corn (*Zea mays* L.)	Leaves	Anti-fluorescein antibody-coated red fluorescent microspheres	Fluorescein (mock pathogen for the proof of concept)	Biotic stress	[34]
*Arabidopsis thaliana* L. Columbia-0	14-day-old seedlings (in vivo)	Fluorescent probe flubactin	ABA receptor PYR1	Abiotic (salinity: 0, 50, 100, 150 μM NaCl)	[31]
*Malus* *hupehensis*, *Malus* × *domestica* cv. Orin, *Nicotiana* *benthamiana*	Seedlings, calli and leaves (in vivo)	Fluorescent probe 2E2F (H_2_O_2_-sensitive trialkylamine merged with aggregation-induced emission fluorescent tetraphenylethene skeleton)	H_2_O_2_	Abiotic (mechanical damage, salinity, high light, drought)	[33]
In silico	In silico	Engineered *Escherichia coli* with abscisic acid receptor PYR1 and luciferase enzyme	ABA	Biotic and abiotic	[32]

### 3.3. Fluorescence-Based Optical Biosensors Expressed by Transgenic Lines

Genetically encoded biosensors (Table 3) are biosensors based on specialized fluorescent proteins designed to alter their light signals when they detect specific physiological changes. As they are protein-based, targeting signals can be attached, directing the sensors to precise locations within a cell, such as the mitochondria or the nucleus. By allowing the real-time monitoring of cellular compartments, the creation of these tools has revolutionized the understanding of cellular physiological conditions [36]. A nuclear-localized FRET-based (Förster Resonance Energy Transfer) biosensor designed to measure the concentration and dynamics of ABA was used to quantify its accumulation at the cellular level during spider mite infestation [37]. A FRET-based biosensor (PAleon), genetically encoded, was specifically designed to monitor the concentration and spatio-temporal dynamics of the signaling lipid phosphatidic acid (PA) in living plant cells and tissues. Indeed, the researchers observed that PA serves as a pH sensor and that salt stress, mannitol, and ABA all trigger rapid PA accumulation [38]. Additionally, Pt-GFP, a genetically encoded fluorescent ratiometric pH sensor, was used to observe the pH variation in response to temperature (cold and heat stress) in potato plants [39]. Heat stress was also evaluated using DREB2A, where DREB stands for Dehydration-Responsive Element-Binding, a temperature-sensing promoter that controls the expression of a yellow fluorescent protein reporter [40].

In addition to the previously described target molecules considered for stress response, Ca^2+^ serves as a cellular messenger that allows plants to perceive and respond to the environment and acts as a proxy for stress. Its dynamics generally integrate with other signaling pathways, such as cellular pH changes and the production of ROS, to coordinate and respond to challenging conditions. The green biosensor GCaMP, a genetically encoded Ca^2+^ indicator (GECI), constituted by calmodulin and GFP, was fused with the red fluorescent dTomato to evaluate the effect of salt stress on *Arabidopsis* roots [41]. Biotic stress is also monitored in vivo and in real-time by tracking the signaling triggered by aphid feeding through the expression of the Ca^2+^ sensor GCaMP3 [42]. Another GECI, the recombinant protein apoaequorin, was expressed in transgenic potato lines. This bioluminescence-based indicator allowed for the quantification of stimulus-specific Ca^2+^ signatures in response to stressors, like salt, osmotic shock (mannitol), and pathogen-associated molecular patterns [43].

The glutathione redox potential is a key proxy for stress in plants, reflecting the balance between reduced (GSH) and oxidized (GSSG) forms [44]. It can be exploited as a biomarker because it integrates various stress signals (e.g., heat, heavy metals, anoxia, pathogen attacks) into a measurable change in oxidative shifts. The green fluorescent protein roGFP, which is sensitive to redox environments and linked to glutaredoxin (GRX), was used to assess the effect of abiotic and biotic stress. In three of the listed studies, it was demonstrated that the biosensor responds ratiometrically to dynamic cellular redox changes [36,44,45].

**Table 3:** Optical biosensors made of genetically encoded fluorescent probes, applied to herbaceous species.

Plant Species	Plant Tissue	Biosensor	Target	Stress	Reference
Corn (*Zea mays* L. line Q319)	Protoplasts	Transgenic corn plants expressing reduction–oxidation-sensitive (roGFPs)	Cellular redox changes	Abiotic (oxidative: 10, 2.5 mM Dithiothreitol; 2.5, 5, 10, 100 mM H_2_O_2_)	[44]
*Arabidopsis thaliana* L. Columbia-0	Leaves (in vivo)	Transgenic *Arabidopsis* plants expressing a Yellow Fluorescent Protein bioreporter under the control of a temperature-sensing promoter	Dehydration-responsive element-binding protein 2A (DREB2A)	Abiotic (high temperature: 38 °C)	[40]
*Arabidopsis thaliana* L. Columbia-0	Leaves and 4-week-old plants (in vivo)	Transgenic *Arabidopsis* plants with fluorescent calcium biosensor (GCaMP3)	Ca^2+^	Biotic (aphid feeding)	[42]
*Arabidopsis thaliana* L. Columbia-0	Roots and seedlings (in vivo)	Transgenic *Arabidopsis* plants with FRET-based phosphatidic acid-specific optogenetic biosensor	Phosphatidic acid	Abiotic (salinity: 100 mM NaCl)	[38]
Potato (*Solanum tuberosum* cv. Nevsky)	Leaves, stems, roots (in vivo)	Transgenic potato plants expressing a fluorescent ratiometric pH sensor *Pt*-GFP	pH	Abiotic (high temperature: 25, 30, 35, 40 and 45 °C)	[39]
*Arabidopsis thaliana* L. Columbia-0	Leaves (in vivo)	Transgenic *Arabidopsis* plants expressing biosensors roGFP2-Orp1 and GRX1-roGFP2 targeted to various organelles	H_2_O_2_ and glutathione redox potential	Biotic (Flg22 from *Pseudomonas syringae* flagellin)	[45]
Barley (*Hordeum vulgare* L. cv. Golden Promise Fast)	Leaves, roots and shoots (in vivo)	Transgenic barley plants with cytosolic redox biosensor Grx1–roGFP2	Glutathione redox potential	Abiotic (salinity: 150 mM NaCl, water potential: −0.8 MPa)	[36]
*Arabidopsis thaliana* L. Columbia-0	Leaves (in vivo)	Transgenic *Arabidopsis* plants expressing nuclear-localized ABA FRET biosensors	ABA	Biotic (2-spotted spider mite *Tetranychus urticae*)	[37]
Potato (*Solanum tuberosum* cv. Désirée) and *Arabidopsis thaliana* L. Columbia-0	Leaves (in vivo)	Transgenic potato and *Arabidopsis* plants with constitutive expression of apoaequorin	Ca^2+^	Biotic (Flg22 from *Pseudomonas syringae* flagellin, Pep-13 from *Phytophthora infestans* glycoprotein) Abiotic (salinity: 0–800 mM NaCl, osmotic: 0–400 mM mannitol, oxidative: 0.100 mM H_2_O_2_)	[43]
*Arabidopsis thaliana* L. Columbia-0	Roots and protoplasts	Transgenic *Arabidopsis* plants with Ca^2+^ ratiometric sensor (green GCaMP6s and red dTomato fluorescent proteins)	Ca^2+^	Abiotic (salinity: 200 mM NaCl)	[41]

Despite the advantageous aspects of using a model organism like *Arabidopsis* to develop these biosensors, there are many problems linked to their translation to other plant species due to differences in genetics, evolution, regulatory mechanisms, physiology, anatomy, and technical feasibility. For instance, while one-to-one orthologs (genes sharing a common ancestor) are a powerful translational resource, evolutionary processes frequently lead to deviations from this relationship, a problem worsened by errors in genome annotation [46]. Many plant species have significantly larger genomes than the compact *Arabidopsis* one, which has led to evolutionary innovations in transcriptional regulation that are not present in the model species. Moreover, direct translation is further limited when considering species with anatomical traits or structures that are not present in *Arabidopsis* [47].

These problems are worsened when translating scientific research into woody plants. When considering trees, which are defined by large size, long lifecycles, and specialized secondary growth, the direct application of *Arabidopsis*-derived mechanisms often results in lost insights. It is indeed possible to fail to understand or observe processes or mechanisms that are fundamental to trees but absent or functioning differently in *Arabidopsis*, and vice versa. Genetic transformation of woody plants is still a time-consuming process, in particular for species with long growth cycles. Their large genomes make these processes even more complex, with efficiency as low as 1% in recalcitrant species, with the best scenario found in Poplar, with transformation frequencies greater than 50% [48].

## 4. Biosensors to Assess Stress in Woody Plants

It is interesting to notice that the selected articles are extremely recent, ranging from 2021 to 2025, further indicating that the topic is still emerging and has been highly attractive in recent years (Table 4). Species used in these studies belong to the Angiosperm clade, while Gymnosperms are not considered. This is possibly explained by the general interest in agronomically relevant species (e.g., apple trees, citrus trees), with eight out of eleven articles focused on these plants, and the remaining three concerning commercially important forest trees such as Poplar. Nonetheless, nine different orders and families are represented in these articles, suggesting that the research in this field is not limited to a dominant tree species. Moreover, the biosensors developed in these studies are generally applicable to different species, creating a system that could be easily scaled to other plants.

Within tissues analyzed, leaves proved to be the main target in seven articles, followed by fruits (four articles), and finally xylem, which was used in only one paper. This inclination for leaves should not be unexpected, considering that they are a preferred organ for many studies due to their central role in fundamental biological processes (photosynthesis, gas and water exchange), their function as environmental indicators, and their practical accessibility for sampling.

Several biosensors were developed for in vivo measurements. To measure glucose levels, Diacci and colleagues developed an implantable organic electrochemical transistor system designed for in vivo, real-time monitoring of plant xylem sap. The device is produced on a flexible polyethylene naphthalate foil for easy insertion into tree stems with minimal invasiveness. The sensors were implanted into the mature xylem of hybrid aspen trees, allowing for continuous data collection for up to 48 h [49]. Salicylic acid (SA) is utilized to evaluate plant stress because it functions as a vital phytohormone and a key signaling molecule that coordinates how plants respond and adapt to both biotic and abiotic stressors, making its concentration fluctuations a reliable indicator of a plant’s health status [50]. A wearable, non-destructive biosensor designed for real-time, in vivo monitoring of SA in plants was developed. The device was specifically engineered to track plants’ physiological response to drought and salinity without wounding the tissue, using a reverse iontophoresis system [50]. For H_2_O_2_ detection, in addition to the fluorescent probe described in chapter 3.2 [33], a different approach resulted in a spray that encapsulates HRP and 2,2’-azino-bis(3-ethylbenzothiazoline-6-sulfonic acid), ABTS, within a metal–organic framework directly on the surface of plant leaves, with a color-to-thermal signal conversion [51].

Other biosensors have been developed for in vitro applications with plant samples (leaves, fruit, extracts). For instance, a hybrid electrode material composed of thionine and nanosheets was designed to overcome the limitations of traditional H_2_O_2_ detection methods, which often require time-consuming sample preparation and specialized laboratory equipment [52]. Being one of the major scavenging enzymes of H_2_O_2_, ascorbate peroxidase (APX) was targeted as a reliable molecular indicator of oxidative stress [53]. The device utilizes a screen-printed carbon electrode modified with gold nanoparticles and anti-APX nanobodies. Because the APX protein sequence is highly conserved, the nanobodies were able to detect the enzyme in leaf extracts from both pea (*Pisum sativum*) and poplar (*Populus nigra*), suggesting the possible application of the biosensor to a broad range of plant species [53]. Isoprene is a valuable stress assessment tool because it acts as a real-time signal of the plant’s internal defense system against abiotic and biotic threats; the amount of isoprene produced increases proportionally with the severity of the stress the plant experiences [54]. Liu and colleagues [55] developed a sensitive coumarin-based fluorescent probe, specifically designed for the real-time detection and imaging of isoprene in plants. Aside from its successful functioning, the detection and imaging process is currently confined to a laboratory setting due to several requirements (fluorescence microscopy, chemical solvents).

The detection of specific biological or chemical stressors that threaten crop productivity and food safety was the focus of four articles. Kazemzadeh-Beneh and colleagues developed a highly sensitive electrochemical DNA probe biosensor for the early detection of *Candidatus Liberibacter asiaticus*, the bacterium that causes citrus huanglongbing, also known as citrus greening disease. The mechanism is based on the DNA hybridization event between the immobilized probe (single-stranded oligonucleotide) and the target DNA sequence from the pathogen [56]. The system could function as an in situ tool for accurate and rapid field screening of the disease. Stigmasterol, a secondary metabolite that serves as a biomarker for oil palm trees infected by the fungus *Ganoderma boninense*, was assessed using a sensitive electrochemical biosensor. This fungus causes lethal diseases like basal stem rot, and the sensor provides a non-enzymatic alternative to complex traditional methods like GC-MS for early disease diagnosis, exploiting screen-printed carbon electrodes modified with β-Cyclodextrin as the molecular recognition element. The study confirmed that stigmasterol levels significantly increased in plants infected with *G. boninense* as part of their natural defense mechanism, allowing the sensor to effectively discriminate between healthy and diseased trees [57]. Gan and Wang developed an enzyme-based colorimetric biosensor specifically designed for the portable and sensitive detection of D-limonene, a volatile organic compound that serves as a critical biomarker for plant health and fruit quality. The authors demonstrated the biosensor’s utility by monitoring D-limonene release during mango post-harvest storage and identifying citrus pest infestations by fruit flies, offering a user-friendly and cost-effective alternative to laboratory instruments like GC-MS [58]. Miglione and colleagues developed an electrochemical inhibition biosensor specifically designed for the rapid, on-site detection of organophosphorus pesticides by simply scrubbing the surface of a fruit with a sensor strip integrated into a glove and performing a reading with a portable potentiostat. The system acts as a tool for precision agriculture, enabling immediate decision-making regarding food safety and pesticide application without the need for laboratory equipment or specialized training [59].

**Table 4:** Biosensors applied to woody plants.

Plant Species	Plant Tissue	Biosensor	Target	Stress	Reference
Hybrid aspens (*Populus tremula* × *tremuloides*)	Mature xylem (in vivo)	Organic electrochemical transistors on polyethylene naphthalate substrate with platinum nanoparticles, GOX, invertase	Glucose, sucrose	Biotic and abiotic	[49]
Kiwi (*Actinidia chinensis* cv. Hongyang)	Leaves (in vivo)	Spray solutions of ABTS, HRP, zeolite imidazolate frameworks-8	H_2_O_2_	Biotic and abiotic (mechanical wounding)	[51]
*Malus* *hupehensis*, *Malus* × *domestica* cv. Orin, *Nicotiana* *benthamiana*	Seedling, calli and leaves (in vivo)	Fluorescent probe 2E2F (H_2_O_2_-sensitive trialkylamine merged with aggregation-induced emission fluorescent tetraphenylethene skeleton)	H_2_O_2_	Abiotic (mechanical damage, salinity, high light, drought)	[33]
Orange (*Citrus* × *sinensis* L.)	Fruit juice	Glassy-carbon electrode, thionine and Ti_3_C_2_T_x_ nanosheets	H_2_O_2_	Biotic and abiotic	[52]
Orange (*Citrus* × *sinensis* L.)	Midribs and fruit peels	Gold electrode with thiolated outer membrane protein-probe	Outer membrane protein of *Candidatus Liberibacter asiaticus*	Biotic	[56]
Oil palm tree (*Elaeis guineensis* Dura × pisifera AVROS)	Leaf extract	Screen-printed carbon electrode with β-cyclodextrin, functionalized, reduced graphene oxide–gold nanoparticles	Stigmasterol	Biotic (*Ganoderma boninense* infection)	[57]
Poplar (*Populus* sp.), willow (*Salix* sp.), birch (*Betula* sp.), clove (*Syzygium aroma ticum*), locust (*Robinia pseudoacacia*)	Leaves	Coumarin fluorescent probe with maleimide	Isoprene	Abiotic (high CO_2_ concentration: 380, 600 ± 10 mol mol^−1^)	[55]
Avocado (*Persea americana Hass*)	Leaves (in vivo)	Laser-induced graphene electrode with polyimide	SA	Abiotic (drought, salinity: 5, 15, 50 mM NaCl)	[50]
*Populus nigra* PN6 clone	Leaf extracts	Amperometric with screen-printed carbon electrode, gold nanoparticles, nanobodies anti-APX	Ascorbate peroxidase	Abiotic (heavy metals: 1850 mg kg^−1^ Pb, 3830 mg kg^−1^ Zn, 21 mg kg^−1^ Cd)	[53]
Mango (*Mangifera* sp.) Citrus (*Citrus reticulata* Ai Yuan 38)	Fruits	Colorimetric with acetylcholinesterase on a zinc-based metal–organic framework	D-limonene	Biotic and abiotic	[58]
Apple (*Malus domestica*), Orange (*Citrus* × *sinensis* L.)	Fruit peels	Screen-printed polyester electrodes with Prussian blue, butyrylcholinesterase, butyrylthiocholine, Dichlorvos	Organophosphorus pesticide	Abiotic	[59]

The application of biosensors to woody plants represents a growing research field, with the expectation that it will develop further in the near future. Overcoming the difficulties linked to the development of new technology, the hope is that this tool can achieve widespread adoption.

## 5. Perspectives

### 5.1. What Plant Species and Plant Tissues Can Be Targeted by Biosensors?

Biosensors were applied to a vast array of plants, ranging from laboratory model organisms and crops to woody forest trees and ornamental species. The use of model plant organisms is strategic because they allow for rapid and cost-effective experimentation due to their short generation times, production of large offspring, small size, and ease of genetic manipulation. Arabidopsis was used in nine of the listed articles, and other commonly used plant model organisms (corn, rice, soybean, tobacco) were the subjects of the other nine articles. This indicates that research in biosensor technology is still in the preliminary phase of concept development (generally with a Technology Readiness Level between 3 and 4), taking advantage of the deeper scientific knowledge available for these species. One of the advantages of some of the biosensors described is that they can potentially be used in any species of interest.

Applying biosensors to woody plants presents unique engineering challenges due to their long-life cycles, complex vascular structures, and robust defense mechanisms. To move these technologies toward practical field use, several core engineering hurdles must be addressed. Firstly, it is fundamental to avoid significant direct interferences between biosensors and plant organs and tissues. Among the solutions, microneedle or flexible film substrates allow minimal trauma, while reverse iontophoresis or spray-on biosensors represent non-invasive methods. The plant’s natural wound response is the most significant barrier to long-term monitoring in woody plants; efforts must focus on materials and surface coatings that specifically delay or minimize cork tissue repair to extend the monitoring timeframe. Lastly, for continuous measurements, biosensors must maintain signal integrity in a complex biological matrix containing molecules that can passivate the sensing interface. To cope with this problem, non-active areas can be encapsulated to ensure that the sole sensing site is exposed to the plant environment, a physical filter can be placed between the plant and the electrode to allow small target molecules to diffuse through or enzymes can be integrated into metal–organic frameworks to protect them from adverse medium conditions or degradation.

To build an effective plant diagnostic system, the primary challenge is identifying the most representative tissue for sampling or for in vivo assessment, specifically, the part of the plant that best reflects physiological shifts caused by external stress. Because the leaf is the hub of metabolism and systemic signaling, it remains the gold standard for diagnosing nutrient status and general environmental perturbations. However, for stressors that originate below ground (heavy metal toxicity, severe drought, high-salinity soil, pathogen infections), analyzing root tissues is essential to fully grasp the physiological decline of the plant.

### 5.2. Which Type of Biosensor to Select?

Biosensors are categorized into various types based on their detection mechanisms, each offering distinct advantages and facing unique challenges when applied to plant systems. The advantages and disadvantages of choosing either electrochemical or optical systems are summarized in Table 5.

The economic feasibility of developing a biosensor for plant use is highly dependent on the transduction mechanism and its application. Electrochemical biosensors offer a cost-effective solution for real-time monitoring, with unit costs ranging from approximately 10 to 10,000 USD depending on the complexity of the potentiostat (commercially available or developed using open-source tools) and electrode materials.

In contrast, biosensors that are developed using optical systems are usually limited by the need for expensive laboratory equipment (such as confocal microscopes or specialized imaging systems), even though some advanced phenotypic probes allow for naked-eye detection using only simple UV light. Additionally, injected fluorescent probes incur higher operational costs, reaching 100–500 USD per reagent vial. One limitation of this system is that it is single-use; once the probe is injected, it eventually photobleaches or is metabolized by the plant, indicating that there is a continuous monitoring demand for the reinjection of the probe. While genetically encoded transgenic lines require substantial initial capital investment in research and development, they represent the most scalable model, as the sensing capability is biologically replicated through subsequent generations at a negligible marginal cost. It is of note, however, that according to the legislation, the cost of securing government permits for Genetically Modified Organisms can reach millions of dollars in legal and environmental impact studies.

### 5.3. The Utility of Biosensors

Scientists should invest in biosensor research because these tools represent a revolutionary shift from traditional destructive snapshot analysis to real-time, in vivo monitoring of biological processes. Unlike laboratory methods that require tissue sampling and complex off-site processing, biosensors allow for the continuous tracking of metabolites and physiological signals directly within living plants, providing unprecedented spatiotemporal resolution. Biosensors can detect biochemical indicators of abiotic and biotic stress well before phenotypic symptoms like yield loss, wilting, or tissue necrosis become visible. This capability allows for timely decision-making and intervention, such as targeted pesticide application or optimized irrigation, which can prevent irreversible damage and significant crop losses. Among the biosensors developed in the listed papers, point-of-care devices, spray-on sensors, and even on-glove sensors were described, allowing farmers and forest services to test for pesticides or diseases directly in the field without specialized training or complex equipment. Minimally invasive technologies like hollow microneedles and reverse iontophoresis allow for the extraction and analysis of plant fluids with minimal damage to the host, preserving the integrity of the plant’s natural responses. For agricultural management, these biosensors can be interfaced with computer-controlled feedback systems (in greenhouses) to automatically regulate the environment based on the plant’s actual physiological state. Integration with AI-driven predictive models further enhances this by providing actionable big data for intelligent farming.

However, some general considerations should be kept in mind. The accuracy of biosensors can be affected by fluctuating external factors like temperature, pH, and humidity, necessitating frequent calibration or mathematical adjustments to ensure reliable data. Mechanical durability and stability can be challenging to maintain, particularly for wearable biosensors. Moreover, even minimally invasive sensors that pierce the plant can trigger wound responses, such as the formation of cork tissue, which may eventually isolate the sensor from the plant’s vascular system and limit the duration of monitoring.

### 5.4. On What Should We Focus Our Efforts?

For the development of a biosensor that can recognize when a plant is subjected to a stressor, it is fundamental to consider the correct biological marker. These markers can be linked to the hormonal metabolism, the oxidative metabolism, or the vast array of secondary metabolites and protein-level changes that regulate cellular homeostasis and defense responses. Selecting an appropriate and stress-specific marker is paramount to ensure the biosensor provides accurate, timely, and relevant data on the plant’s condition. To note, generally, the mechanisms activated in response to stress are mutually interconnected or associated with other signaling pathways, creating an intricate network. Additionally, these responses are not fully deciphered or conserved in all plant species. Therefore, the selection of the correct marker is a task that should not be underrated, as is the interpretation of the results obtained. As mentioned before, when a plant is subjected to a stressor, either biotic or abiotic, it might trigger a dose-dependent response, according to the hormesis theory [60]. At lower concentration the plant can respond in a positive manner, acting as a biostimulant (e.g., increasing productivity, growth, and yield quality). However, when the dose exceeds a certain threshold, negative and toxic responses arise, activating mechanisms that can have deleterious (but possibly reversible) or lethal (irreversible) effects. In addition to the dose concentration, the plant reaction also depends on the duration of the exposure to stress conditions.

Ideally, the target molecule chosen that acts as a proxy for plant stress should represent early-stage stress conditions, generally undetectable by standard visual assessment, allowing for effective intervention to safeguard plant health. Moreover, the possibility of assessing when the stressor threshold has been passed could allow for prompt action to safeguard the plant or recognize and predict delayed tree mortality events.

In the literature, several promising physiological indicators have been identified as successful tools for diagnosing stress across various species, including relative water content, photosynthetic markers, membrane integrity, and antioxidant defense. Nonetheless, the sensitivity of these markers often depends on the specific stressor (drought, salinity, extreme temperatures), making it mandatory to first optimize diagnostic protocols for a given species–stressor combination. Because redox metabolism is deeply integrated into systemic signaling and homeostatic regulation [61], the use of redox markers, specifically those related to oxidative stress and antioxidant activity, represents a highly promising strategy for establishing robust indicators of a plant’s stress state. As confirmation, H_2_O_2_ resulted in one of the most recurrent targets found for biosensor development (8 out of 35 research papers). The second most common target was hormones (6 out of 35 research papers). This should come as no surprise since they act as versatile chemical regulators that coordinate almost every aspect of growth, development, and environmental response. Plants use hormones as signaling molecules to control internal activities and transfer stress responses between different parts of the organism. This allows the plant to coordinate a systemic response even if the stressor is localized to a specific tissue. More importantly, hormone levels often change well before phenotypic manifestations of damage (such as wilting, necrosis, or yield loss) appear. Monitoring these fluctuations allows for timely interventions to boost resilience and productivity. Moreover, glucose biosensors, being one of the most famous examples of this technology, with their well-studied and developed electrochemical system using GOX, remained undervalued, with only four articles focusing on this metabolite. This compound could instead represent a good starting point; it is involved in signaling processes linked to different stressors, nutrient deficiency, osmotic, drought, temperature, and oxidative [62], making it applicable to a plethora of environmental conditions.

## 6. Outlooks and Conclusions

Plant biosensors have a diverse range of potential applications in modern agriculture, environmental monitoring, and basic plant science. Future efforts should be directed toward moving these technologies from controlled laboratory settings to practical field conditions. For agricultural applications, biosensors could detect early biochemical indicators of drought, salinity, and water stress before physical symptoms appear. This allows for optimized irrigation strategies and timely interventions to reduce crop loss. The same concept is applied to early pathogen and disease detection (in planta and for post-harvest quality and storage), integrating systems for notification at the first onset of infection, rather than waiting for visible indicators. With a view to automated agricultural systems, biosensor outputs can be linked to computer-controlled feedback loops in greenhouses to automatically regulate environments (light, temperature, humidity) based on the actual physiological state of the plant. As for forestry, given that trees have long life cycles and are of vital importance for global CO_2_ fixation, the priority should be the development of technologies that can detect stress before phenotypic decline, allowing for large-scale forest monitoring and precision management. A significant challenge in tree-based biosensing is the plant’s natural defense against disturbance. Implantable sensors, such as organic electrochemical transistors in xylem tissue, currently trigger wound responses like cork tissue formation within a few days. To extend the duration of real-time monitoring, future efforts should be directed toward engineering sensor designs that minimize this response. While current bioelectronic sensors in trees provide valuable insights into relative changes, they are often limited to qualitative observations. A major objective is the development of internal reference systems that will enable the absolute quantification of metabolites within living woody tissues, providing a deeper understanding of the allocation and transport of target compounds.

The next generation of biosensors should be integrated into a digital infrastructure for AI management: algorithms should be used to optimize sensor parameters and analyze complex datasets to predict plant health trends. Real-time data from sensors could be linked to big data platforms, enabling remote, long-term monitoring of physiological changes and contaminant levels. Future devices should aim for the simultaneous detection of multiple analytes (e.g., different hormones and metabolites) to provide a more comprehensive understanding of complex stress responses. Moreover, they should convert biochemical signals into values displayed directly on a smartphone screen or handheld device, facilitating immediate decision-making for farmers and forest services. Finally, much research currently relies on easily transformable model plants like Arabidopsis. Efforts should shift towards applying these tools to major staple crops (wheat, maize, rice) and forest trees: considering their long-life cycles and acting as primary engines for global CO_2_ fixation, they represent a multi-generational investment in climate stability.

## Figures and Tables

**Table 5:** Advantages and disadvantages of the three types of electrochemical or optical biosensors.

Biosensor Type	Advantages	Disadvantages
Electrochemical	High sensitivity and high specificity [24,29,52,56]	Susceptible to background interference and matrix effects from complex biological fluids [24,56]
	Fast response times with real-time, in vivo applications; provide a continuous, numerical data stream easy to digitize and process [26,28,52,59]	Plant compounds like ascorbic acid or uric acid can interfere with current readings [24]
	When using platforms like microneedle arrays, they enable minimally invasive analysis causing negligible damage [24,25,26]	Long-term monitoring is often limited by plant’s wound response [49]
	Technologies like screen-printing and 3D printing make them cost-effective and scalable for mass production [19,24,53,57]	
	Prone to miniaturization and integration into portable devices [24,53,56,59]	
Optical: injected fluorescent probe	Provide ultra-high spatial and temporal resolution for real-time tracking of biomarkers [33,55]	May lack specificity; some probes react with a broad range of molecules rather than a single target [45]
	Offer simple operation and low cost once the probe is developed [33,55]	Highly susceptible to photobleaching and photodamage, limiting continuous observation [32,39]
	Work on any species, including those that are hard to genetically transform [33,55]	Signal accuracy is hindered by chlorophyll autofluorescence, creating background noise [51]
	Usually present strong biocompatibility, allowing for the study of intact in vivo samples [33,55]	Exogenous addition of probes may cause toxicity to the plant [39]
		Solutions can be difficult to infuse through plant surfaces and distribution can be uneven [51]
Optical: transgenic fluorescent line	Ratiometric measurements, making the readout independent of the specific level of probe expression [36,44]	Limited to readily transformable model plants, application to major agricultural crops or woody trees is extremely laborious, time-consuming [31,35]
	Continuous, real-time monitoring of physiological dynamics [39,44]	Readouts can be obscured by background signals from plant compounds [35,36]
	Targeted to specific subcellular compartments (cytosol, nuclei, mitochondria) to study localized stress responses [36]	Require external excitation light sources and are susceptible to photobleaching [32,35]
	Non-invasive, once the plant is grown, no further wounding is needed [39,44]	Expression of the sensor protein can cause visible physiological defects, acting as a metabolic burden [45]

**Figure A1:**
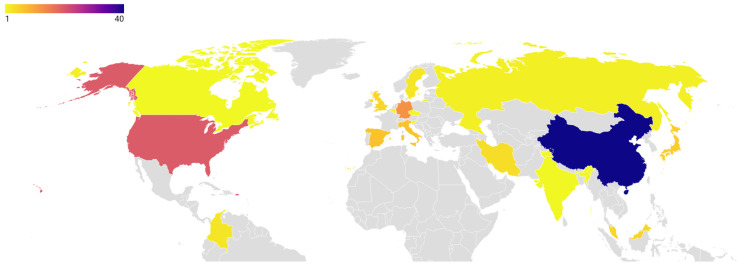
Schematic map of the global distribution of the affiliations of the selected papers. Colors indicate the number of affiliations in the gradient: from light yellow (1) to blue (40). States in gray did not present any affiliations. The figure was created with Datawrapper (datawrapper.de).

## Data Availability

No new data were created or analyzed in this study. Data sharing is not applicable to this article.

## Supplementary Materials

The following supporting information can be downloaded at: https://www.mdpi.com/article/10.3390/bios16050242/s1, Table S1: PRISMA 2020 Abstracts checklist; Table S2: PRISMA 2020 checklist.

## Abbreviations

The following abbreviations are used in this manuscript:

ABA	Abscisic acid
ABTS	2,2’-azino-bis(3-ethylbenzothiazoline-6-sulfonic acid)
APX	Ascorbate peroxidase
FRET	Förster resonance energy transfer
GC-MS	Gas chromatography–mass spectrometer
GECI	Genetically encoded Ca^2+^ indicator
GFP	Green fluorescent protein
GOX	Glucose oxidase
HPLC	High-performance liquid chromatography
HRP	Horseradish peroxidase
IAA	Indole 3-acetic acid
PICO	Problem, intervention, comparison, and outcome
ROS	Reactive oxygen species
SA	Salicylic acid

## Appendix A. Additional Information on the PICO Strategy for the Selection of the Research Papers

For herbaceous plants the search query was defined as:(stress*) AND (biosensor) AND (plant* specie* OR crop), while for woody plants the following keywords were chosen: (environmental stress* OR biotic stress* OR abiotic stress* OR stress) AND (electrochem* biosens* OR biosens* OR sensor*) AND (plant* specie* OR tree* specie* OR woody specie* OR forest). In both cases, the research was conducted within the field “Article Title, Abstract, Keywords”. A time filter, with the range 2010–2025, was applied to screen the results for the most recent ones; moreover, only research papers were considered, avoiding books, chapters, and reviews.

For the herbaceous plants, from the initial 3121 papers found in both Scopus and Web of Science, accessed, respectively, on 25 August 2025 and 10 September 2025, only 25 were found to reflect the selected parameters.

For the woody plants, the starting point consisted of 3156 research papers from Scopus (accession date: 2 July 2025) and Web of Science (accession date: 8 July 2025). Due to the specificity of the argument chosen and the restricted number of articles that were selected according to the fixed parameters, six in total, the search was extended to Google Scholar. In this case, the selective process was conducted for the first 2000 results, obtaining another five results that were considered for the review, reaching a total of 11 articles.

The distribution of the authors’ affiliations of the selected papers (Figure A1) demonstrates that research on plant biosensors is a global effort, with a major distribution in East Asia, North America, and Europe, along with emerging contributions from South America and the Middle East. China was the main contributor with 33% of affiliations, followed by the USA (16%) and Germany (11%).
